# Cytochrome oxidase subunit 2 gene allows simultaneous detection and typing of *Trypanosoma rangeli* and *Trypanosoma cruzi*

**DOI:** 10.1186/1756-3305-6-363

**Published:** 2013-12-23

**Authors:** Amanda Regina Nichi de Sá, Mário Steindel, Lara Maria Kalempa Demeu, Débora Denardin Lückemeyer, Edmundo Carlos Grisard, Quirino Alves de Lima Neto, Silvana Marques de Araújo, Max Jean de Ornelas Toledo, Mônica Lúcia Gomes

**Affiliations:** 1 Departamento de Ciências Básicas da Saúde, Universidade Estadual de Maringá (UEM), Av. Colombo, 5790, Zona 7. CEP: 87020-900, Maringá, Paraná, Brazil; 2 Departamento de Microbiologia, Imunologia e Parasitologia, Universidade Federal de Santa Catarina (UFSC), FlorianópolisSanta Catarina, Brazil; 3 Departamento de Biologia Celular e Genética, Universidade Estadual de Maringá (UEM), Paraná, Brazil

**Keywords:** *Trypanosoma rangeli*, *Trypanosoma cruzi*, Cytochrome oxidase subunit 2 gene, Diagnosis, Typing

## Abstract

**Background:**

The parasites *Trypanosoma rangeli* and *Trypanosoma cruzi* share vectors and hosts over a wide geographical area in Latin America. In this study, we propose a single molecular approach for simultaneous detection and typing of *T. rangeli* and *T. cruzi*.

**Methods:**

A restriction fragment length polymorphism analysis of the mitochondrial cytochrome oxidase II gene (COII-RFLP) using enzyme *Alu*I and different amounts of DNA from the major genetic groups of *T. rangeli* and *T. cruzi* (KP1+/KP1- and DTU-I/DTU-II) was carried out. The same marker was tested on the other *T. cruzi* DTUs (DTU-III to DTU-VI) and on DNA extracted from gut contents of experimentally infected triatomines.

**Results:**

The COII PCR generates a ~400 bp fragment, which after digestion with *Alu*I (COII-RFLP) can be used to distinguish *T. rangeli* from *T. cruzi* and simultaneously differentiate the major genetic groups of *T. rangeli* (KP1+ and KP1-) and *T. cruzi* (DTU-I and DTU-II). The COII-RFLP generated bands of ~120 bp and ~280 bp for KP1+, whereas for KP1- no amplicon cleavage was observed. For *T. cruzi*, digestion of COII revealed a ~300 bp band for DTU-I and a ~250 bp band for DTU-II. For DTU-III to DTU-VI, COII-RFLP generated bands ranging from ~310 to ~330 bp, but the differentiation of these DTUs was not as clear as the separation between DTU-I and DTU-II. After AluI digestion, a species-specific fragment of ~80 bp was observed for all DTUs of *T. cruzi*. No cross-amplification was observed for *Leishmania* spp., *T. vivax* or *T. evansi*.

**Conclusions:**

The COII-RFLP allowed simultaneous detection and typing of *T. rangeli* and *T. cruzi* strains according to their major genetic groups (KP1+/KP1- and DTU-I/DTU-II) *in vitro* and *in vivo,* providing a reliable and sensitive tool for epidemiological studies in areas where *T. rangeli* and *T. cruzi* coexist.

## Background

*Trypanosoma rangeli* and *Trypanosoma cruzi* are protozoan parasites that infect sylvatic and domestic mammals and humans in several Central and South American countries [1,2]. *T. rangeli* is considered nonpathogenic to mammals, while *T. cruzi,* the causative agent of Chagas disease, affects around 7 to 8 million people in Latin America, leading to morbidity or mortality [3-5]. Since these parasites share a variety of soluble antigens, vectors, and hosts over a wide geographical area, mixed infections in triatomines and mammals may occur [6,7], reinforcing the need for specific diagnosis [3,4,8-13].

Several nuclear and mitochondrial molecular markers have been used for detection and differentiation of *T. rangeli* and *T. cruzi*[9,14-19], but none of them allows typing of strains according to the currently described genotypes. The sequence variability of the kinetoplast DNA (kDNA) minicircle was used to classify *T. rangeli* strains into two major genetic groups, termed KP1+ and KP1- [20-22], which were further confirmed by others [4,23].

Assessment of *T. rangeli* genetic variability is based on studies of one or a few nuclear or mitochondrial markers [2,23-26], which indicate the intraspecific genetic variability of this parasite and confirm the KP1+ and KP1- genetic groups [21,22].

Differently from *T. rangeli*, the genomic plasticity of *T. cruzi* is well described in the literature [6,8,27-37]. Strains of this parasite have been classified in six discrete typing units or DTUs (DTU-I to DTU-VI), which were further divided into subtypes by several authors [29,38,39], and a recent genotype identified as TcBat [35,36,40].

Restriction fragment length polymorphism (RFLP) analysis of the mitochondrial cytochrome oxidase II (COII) subunit 2 gene, termed COII-RFLP, was originally proposed by Freitas *et al.*[30] and modified by Abolis *et al.*[7] to distinguish *T. cruzi* DTUs isolated from southern Brazil. This method has not yet been used to detect and type *T. rangeli* strains. In this study, considering the sympatric occurrence in mammals and vectors and the genetic plasticity of *T. rangeli* and *T. cruzi*, we used COII-RFLP as a single molecular approach for simultaneous detection and typing of these two parasites.

## Methods

### Parasites

Parasite strains used in this study are shown in Table 1. *T. rangeli* KP1+ and KP1- strains were characterized according to the methodology of Vallejo *et al.*[21]. *T. cruzi* DTUs were the standard strains described by Zingales *et al*. [35,36].

**Table 1:** Trypanosomatid species and strains used in this study, their original hosts, geographical origins and genetic groups

**Species**	**Strain**	**Hosts**	Geographical origin*	Genetic group**
*Trypanosoma cruzi*	**Sylvio X10**	*Homo sapiens*	Brazil (PA)	DTU-I
	**Esmeraldo cl3**	*H. sapiens*	Brazil (BA)	DTU-II
	**231**	*H. sapiens*	Brazil (MG)	DTU-III
	**CAN III**	*H. sapiens*	Brazil (PA)	DTU-IV
	**SO3 cl5**	*Triatoma infestans*	Bolivia	DTU-V
	**CL Brener**	*T. infestans*	Brazil (RS)	DTU-VI
	**150**	*H. sapiens*	Brazil (MG)	DTU-I
	**328**	*H. sapiens*	Brazil (PR)	DTU-II
	**SC-90**	*Didelphis aurita*	Brazil (SC)	DTU-I
	**SC-95**	*H. sapiens*	Brazil (SC)	DTU-II
*Trypanosoma rangeli*	**Choachí**	*Rhodnius prolixus*	Colombia	KP1+
	**H8GS**	*H. sapiens*	Colombia	KP1+
	**San Agostín**	*H. sapiens*	Colombia	KP1+
	**D3493**	*R. prolixus*	Colombia	KP1+
	**H14**	*H. sapiens*	Honduras	KP1+
	**H9**	*H. sapiens*	Honduras	KP1+
	**Macias**	*H. sapiens*	Venezuela	KP1+
	**Palma 2**	*R. prolixus*	Venezuela	KP1+
	**R1625**	*H. sapiens*	El Salvador	KP1+
	**B450**	*Rhodnius robustus*	Brazil (PA)	KP1+
	**1545**	*R. prolixus*	Colombia	KP1+
	**SC-58**	*Echimys dasythrix*	Brazil (SC)	KP1-
	**SC-61**	*E. dasythrix*	Brazil (SC)	KP1-
	**SC-68**	*Panstrongylus megistus*	Brazil (SC)	KP1-
	**SC-74**	*P.megistus*	Brazil (SC)	KP1-
	**SC-75**	*P.megistus*	Brazil (SC)	KP1-
	**C-23**	*Aotus trivirgatus*	Colombia	KP1-
*Trypanosoma evansi*	**Te**	*Canis familiaris*	Brazil (RS)	NA
*Trypanosoma vivax*	**Tv**	*Bos taurus*	Brazil (PB)	NA
*Leishmania amazonensis*	**M2269**	*H. sapiens*	Brazil (PA)	NA
*Leishmania braziliensis*	**M2903**	*H. sapiens*	Brazil (PR)	NA
*Leishmania infantum*	**LRM75**	*H. sapiens*	Brazil (PI)	NA

* Letters in parentheses indicate the Brazilian state where the strain was isolated (BA, Bahia; MG, Minas Gerais; PA, Pará; RS, Rio Grande do Sul; SC, Santa Catarina). ** DTU-I to DTU-VI are the different *T. cruzi* DTUs (Discrete Typing Units) described by Zingales *et al*. [35,36], and KP1+/KP1- are the *T. rangeli* genetic groups described by Vallejo *et al.*[21]. NA –Not Applicable.

### Genomic DNA extraction and quantification

*T. rangeli* DNA was extracted by the standard phenol-chloroform method, and *T. cruzi* DNA was extracted as previously described [41]. DNA from *Leishmania amazonensis*, *Leishmania braziliensis*, *Leishmania infantum*, *Trypanosoma evansi* and *Trypanosoma vivax* was extracted by the standard phenol-chloroform method and used as controls. Extracted DNA was eluted in TE buffer (10 mM Tris–HCl pH 8.0 and 1 mM EDTA pH 8.0), quantified by spectrophotometry at 260/280 nm, and stored at -20°C until use.

### COII-RFLP analysis

Amplification of the COII gene by PCR following restriction fragment length polymorphism analysis with *Alu*I (COII-RFLP) was used to simultaneously detect and differentiate strains of *T. rangeli* (KP1+/KP1-) and *T. cruzi* (DTU-I to DTU-VI).

COII amplification was carried out as described by Freitas *et al*. [30] with modifications proposed by Abolis *et al*. [7]. Briefly, each amplification reaction was performed in a final volume of 15 μL, containing 3.1 pmol of primers Tcmit-10 (5′-CCA TAT ATT GTT GCA TTA TT-3′) and Tcmit-21 (5′-TTG TAA TAG GAG TCA TGT TT-3′), 2 ng of DNA (ideal amount for amplification in relation to the tested amounts, from 0.1 fg to 2 ng), 2.5 μM of dNTP, 3.5 mM of MgCl_2_, and 1 U of Platinum *Taq* DNA polymerase (Invitrogen) in the buffer provided by the manufacturer. Amplification was processed in a Techne TC-512 thermocycler with initial denaturation at 94°C for 1 min, followed by 30 cycles (94°C for 30 s, 48°C for 2 min, and 72°C for 2 min) and a final extension step at 72°C for 10 min. Ten microliters of unpurified amplification product was digested with 10 U (1 μL) of the *Alu*I restriction enzyme (New England BioLabs) in the buffer (1 μL) provided by the manufacturer for 16 h at 37ºC. From artificial mixtures of DNA of the four major genetic groups processed simultaneously in a single reaction, the groups DTU-I, DTU-II and KP1+ were considered an internal control of enzymatic digestion. The resulting fragments were analyzed in 6% polyacrylamide gel, silver-stained and digitally recorded.

### *T. rangeli* COII gene sequencing and comparative sequence analysis

The *T. rangeli* COII gene fragment (~400 bp) amplified by PCR from *T. rangeli* Choachí and SC-58 strains was cloned in the pGEM®-T Easy Vector System plasmid (Promega) and transformed in *Escherichia coli* DH5α competent bacteria by heat shock according to standard protocols. After culture for 16–18 h at 37°C on Luria-Bertani medium (LB), three positive clones were subjected to recombinant DNA extraction by the CTAB (cetyltrimethylammonium bromide) method [42] and checked by PCR for the presence of the insert. The plasmid inserts of the three clones had both strands sequenced using the DYEnamic™ ET Terminator Kit, using M13 primers in a MegaBACE 1000 sequencer (GE Healthcare). The sequences obtained were assembled and analyzed using the Phred/Phrap/Consed package [43] following comparison of all high-quality sequences (Phred > 20) with public databases using the BLAST routine. Sequences of the COII gene from *T. cruzi* Sylvio [GenBank: EU302222.1], Esmeraldo [GenBank: AF359035.1], 231 [GenBank: DQ343720.1], CAN III [GenBank: AF359030.1], SO3 cl5 [GenBank: AF359039.1] and CL Brener [GenBank: AF359041.1] strains were retrieved from GenBank. Multiple alignments of nucleotide sequences were performed using ClustalW software [44], and alignment was trimmed by using the BioEdit software [45]. COII sequences from *T. rangeli* Choachí and SC-58 strains were deposited in GenBank under accession numbers HQ691249.1 and HQ691248.1, respectively.

### Targeting the COII gene: Detection and characterization of *T. rangeli* and *T. cruzi* by PCR

The COII-RFLP for detecting and typing *T. rangeli* (KP1+/KP1) and *T. cruzi* (DTU-I/DTU-II) was tested *in vitro* with DNA of each genetic group and in artificial mixtures of DNA in the ratios 1:1 to 1:9 (corresponding to amounts from 2 ng to 0.02 ng) and *in vivo* in triatomines, simulating natural infections. Groups of ten fifth-instar *Rhodnius prolixus* were experimentally infected using an artificial feeding apparatus, using pure or mixed culture-derived epimastigotes from the *T. cruzi* SC-90 (DTU-I) and SC-95 (DTU-II), *T. rangeli* Choachí (KP1+) and SC-58 (KP1-) strains, in the following combinations: DTU-I or DTU-II alone, KP1(+) and KP1(-) alone, DTU-I + KP1(+), DTU-I + KP1(-), DTU-II + KP1(+), DTU-II + KP1(-), DTU-I + DTU-II + KP1(+), DTU-I + DTU-II + KP1(-), DTU-I + KP1(+) + KP1(-), DTU-II + KP1(+) + KP1(-), and DTU-I + DTU-II + KP1(+) + KP1(-). After 30 to 45 days of the infective meal, the midgut and hindgut of five insects were removed and total DNA was extracted as described by Macedo *et al*. [41]. For the other *T. cruzi* DTUs (DTU-III to VI), the performance of the COII-RFLP assay was not tested with artificial or natural mixed infections.

As an external control to compare the efficiency of COII-RFLP in separating *T. cruzi* DTU-I from DTU-II and *T. rangeli* KP1+ from KP1-, the ribosomal RNA (rRNA) gene from the same samples was amplified, using a multiplex PCR reaction as described by Souto *et al*. [17].

## Results

**Figure 1:**
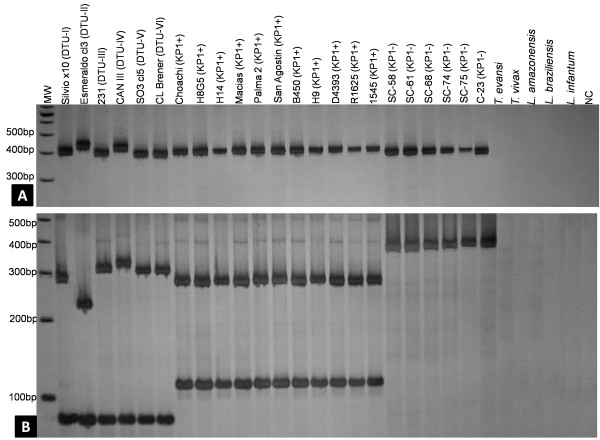
**COII gene assayed from *Trypanosoma cruzi, Trypanosoma rangeli *and other trypanosomatids (Table**1**). A)** COII gene amplification products from *T. cruzi* (lines 1 to 6)*, T. rangeli* (lines 7 to 23); **B)** RFLP pattern obtained by *Alu*I digestion of the cytochrome oxidase subunit II (COII) gene from *T. cruzi* (DTU-I to DTU-VI) strains and *T. rangeli* (KP1+ and KP1-) resolved in 6% polyacrylamide gel stained with silver nitrate. MW = 100 bp DNA Ladder (Invitrogen), NC = Negative control (No DNA added).

Amplification of the COII gene from all *T. cruzi* strains representing the six DTUs and from all *T. rangeli* strains representing the two major genetic groups (KP1+ and KP1-) revealed the expected band of ~400 bp (Figure 1A). Digestion of the amplified fragments with *Alu*I generated the RFLP profile shown in Figure 1B. Regardless of DTU, an 80 bp band was observed for all *T. cruzi* strains; this band was absent in *T. rangeli*. Except for *T. cruzi* DTU-III, DTU-V and DTU-VI, digestion products ranging from 250 to 330 bp allowed the DTU-I, DTU-II and DTU-IV strains to be distinguished (Figure 1B). Digestion of COII amplicon from *T. rangeli* KP1+ strains generated restriction fragments of approximately 125 and 280 bp, while the amplicons from all KP1- strains still consisted of ~400 bp, indicating that they were not digested by *Alu*I (Figure 1B). No amplification of the COII gene was observed for *T. evansi, T. vivax, L. amazonensis, L. braziliensis* and *L. infantum* (Figure 1A, 1B).

**Figure 2:**
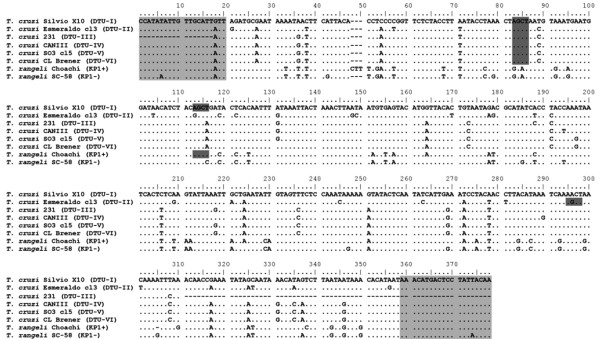
**Alignment of *Trypanosoma rangeli *and *Trypanosoma cruzi *COII gene.** Dots indicate identity; dashes indicate gaps introduced to maximize the alignment of the sequences; bold nucleotides on dark-gray background indicate the *Alu*I restriction sites, and light-gray boxes indicate the annealing sites of primers Tcmit-10 (positions 1–20) and Tcmit-21 (positions 359–378).

The absence of cleavage of the COII gene for *T. rangeli* KP1- strains was further investigated by sequencing and comparative analysis of the amplification products. Multiple sequence analysis was carried out using the COII gene from *T. rangeli* Choachí (377 bp) and SC-58 (375 bp) strains, and *T. cruzi* COII gene sequences retrieved from GenBank. Sequences of the *T. rangeli* COII gene from Choachí (KP1+) and SC-58 (KP1-) strains were 95.2% similar to each other, showing 15 nucleotide substitutions, an insertion (CTT) on positions 48–50 for the Choachí strain, and the absence of the *Alu*I cleavage site in the SC-58 strain (positions 113–116), which was present in the Choachí strain (Figure 2). The inter-specific analysis of COII gene sequences showed similarities between *T. rangeli* and *T. cruzi*. The number of single nucleotide polymorphisms (SNPs) between *T. cruzi* DTU-I and DTU-II strains (90.6% similarity) was twice that observed between *T. rangeli* KP1+ and KP1- strains. *T. rangeli* KP1+ exhibited a similarity of 85.9% with DTU-I and 85.1% with DTU-II, whereas KP1- showed 85.0% similarity with DTU-I and 84.2% with DTU-II.

**Figure 3:**
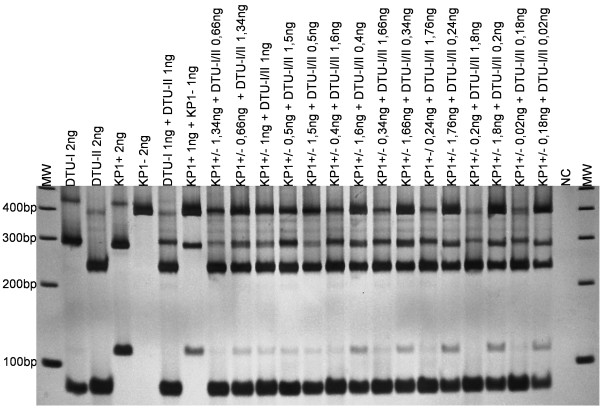
**Profiles of COII-RFLP from DNA of each genetic group of *Trypanosoma cruzi *and *Trypanosoma rangeli *and from artificial mixtures with different ratios of DNA.** Lines 1 to 4 are patterns of PCR-RFLP with *Alu*I in 6% polyacrylamide gel stained with silver from DNA of *T. cruzi* (DTU-I and DTU-II) and *T. rangeli* (KP1+ and KP1-), and lines 7 to 21 are artificial mixtures in the ratios 1:1 to 1:9, with amounts of DNA ranging from 2 ng to 0.02 ng. NC = negative control. MW = 100 bp DNA Ladder.

**Figure 4:**
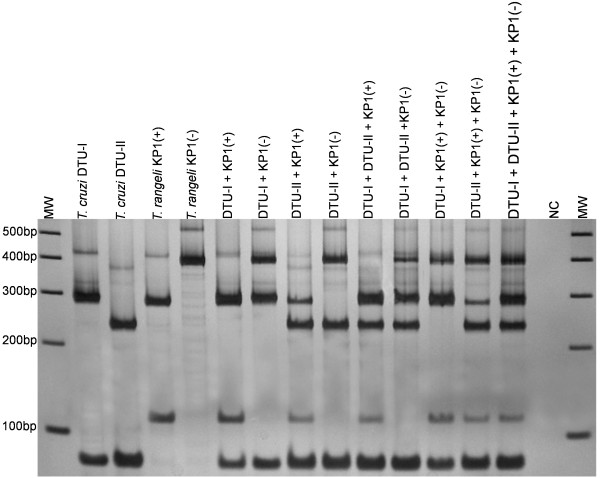
**Profiles of COII-RFLP from *Trypanosoma cruzi *and *Trypanosoma rangeli *in triatomine intestinal tracts.** Silver-stained 6% polyacrylamide gel reveals the electrophoretic profiles of the COII-RFLP assay for detection and typing of *T. rangeli* (KP1+ and KP1- strains) and *T. cruzi* (DTU-I and DTU-II strains) in the intestinal tract of *Rhodnius prolixus* experimentally infected with single or mixed strains and genetic groups. MW = 100 bp DNA Ladder (Invitrogen, USA). NC = Negative control (No DNA added).

The COII-RFLP allowed detection and typing of *T. rangeli* and *T. cruzi* strains in artificial DNA mixtures; as little as 0.02 ng of DNA from both parasites could be detected (Figure 3). For this purpose, detection and typing of parasites present in the intestinal tracts of experimentally infected triatomines were also performed by COII-RFLP, with interesting results. As shown in Figure 4, a clear differentiation between *T. rangeli* and *T. cruzi* was achieved, as well as typing of these parasites according to their major genetic groups. The *T. cruzi* diagnostic band (~80 bp) observed in the COII-RFLP using extracted DNA from each parasite and genetic group was also present in the assay performed with the gut contents from experimentally infected triatomines, revealing the usefulness of the method. Even when *T. rangeli* (KP1+ and KP1-) and *T. cruzi* (DTU-I and DTU-II) are present in the same triatomine, which is commonly observed in nature, it is possible to identify each individual parasite species and genetic group.

The multiplex rRNA PCR was also able to detect and distinguish *T. cruzi* DTU-I (260 bp) and DTU-II (300 bp) strains in both reconstitution tests (artificial DNA mixture) and in the experimentally infected triatomines. For the *T. rangeli* strains, a single band of ~210 bp was observed for both KP1+ and KP1- genotypes (data not shown), in contrast to COII-RFLP, which distinguished these two genetic groups.

## Discussion and conclusions

The COII-RFLP described in this study allows specific detection of *T. rangeli* and *T. cruzi,* even in mixed infections, as well as assessment of their major genetic groups circulating in Latin America, i.e., KP1+/KP1- for *T. rangeli* and DTU-I/DTU-II for *T. cruzi*[46,47]. This assay was also tested using DNA from kinetoplastids of different species (*L. braziliensis*, *L. amazonensis*, *L. infantum*, *T. vivax* and *T. evansi*), with negative results on PCR, confirming that the COII primers are specific for *T. cruzi* and *T. rangeli*. Other *Leishmania* species of medical and epidemiological importance such as those belonging to the *L. guyanensis* complex, as well as *L. naiffi* and *L. lainsoni* remain to be evaluated. However, considering the different organization of the *Leishmania* sp. maxicircles compared to *Trypanosoma* spp. and the results obtained for *L. braziliensis*, *L. amazonensis* and *L. infantum*, we should expect no cross-amplification.

The COII-RFLP, similar to the multiplex PCR targeting rRNA proposed by Souto *et al*. [17], is able to differentiate *T. cruzi* DTU-I, DTU-II and *T. rangeli*, with the additional advantage of distinguishing the parasite’s KP1+ and KP1- genotypes. However, in artificial mixtures where DNA was present in amounts ≤ 0.4 ng, the fragments of ~120 bp from KP1+ showed a lower intensity, indicating that the amount of DNA in the sample, incomplete enzymatic digestion, or competition by enzymes of genetic groups that had larger amounts of DNA may influence the results. The absence of cleavage by *Alu*I for the *T. rangeli* KP1- COII gene was due to a single nucleotide polymorphism (SNP) on the enzyme recognition site, as revealed by high-quality sequencing of the amplicons. This result was confirmed by COII-RFLP performed with artificial mixtures of DNA of the four major genetic groups (DTU-I, DTU-II, KP1+ and KP1-) in a single reaction, where KP1- was the only strain that was not cut by the enzyme. This difference allows a clear distinction of *T. rangeli* KP1+ from KP1- strains in the COII-RFLP analysis. This is also the first analysis of a maxicircle gene from *T. rangeli* kDNA, indicating that both maxi- and minicircles can be used for detection and genotyping strains of this taxon.

Due to the overlapping geographical distribution and the sharing of host and vector species as well as antigens by *T. rangeli* and *T. cruzi*, the existence of mixed infections in mammals and triatomines is a serious problem for diagnosis and epidemiological studies of the two parasites [2,4,11-13]. The COII-RFLP assay described here proved to be able to detect DNA and to assess the genetic groups of both parasites in artificial mixtures *in vitro* and *in vivo* in a single assay on polyacrylamide gel, because the resolution of the agarose gel to discriminate RFLP profiles was so low.

Several studies have successfully developed molecular markers to detect mixed infections, but all of them lack the ability to differentiate the genetic groups of these parasites in a simple and straightforward way, requiring the use of further molecular methods [9,19,48]. The inconsistency of results obtained using different markers, along with the genetic plasticity of both *T. cruzi* and *T. rangeli*, have led several authors to use different methods to achieve proper detection and typing of parasites in natural mixed infections in triatomine bugs [8]. Other investigators have corroborated our findings by showing that COII-RFLP detects mixed infections containing the two major DTU strains of *T. cruzi* (DTU-I and DTU-II) [46,47] isolated from naturally infected triatomines, demonstrating the epidemiological application of this molecular marker [7], but not including the typing of the strains according to their genotypes. Hamilton *et al.*[49] described a fluorescence-based method for typing *T. rangeli* strains; however, this method is laborious, requires the use of DNA sequencers, and considers the existence of five different genetic groups for *T. rangeli* (A-E) [50-52] that are comprised within the KP1+ and KP1- lineages. In summary, *T. rangeli* KP1+ strains from Vallejo *et al.*[21] include strains isolated from Central America, Colombia, Venezuela and Brazil in Group A, whereas KP1- includes Groups B (Brazil), C (Central America, Colombia, Peru), D (southern Brazil) and E (central Brazil) [49-52].

Considering the wide genetic variability, including intra-DTU variation, and the extensive geographic ranges of the parasites studied here, the COII-RFLP was assayed with a limited but representative number of samples. The results showed that compared to the multiplex PCR targeting the rRNA gene, COII-RFLP has a clear advantage in differentiating *T. rangeli* KP1+ and KP1- groups and simultaneously *T. cruzi* DTU-I and DTU-II, the most prevalent genotypes circulating in Central and South America. Thus, COII-RFLP proved to be a simple and useful tool for molecular epidemiology studies in areas where *T. rangeli* and *T. cruzi* are sympatric.

## Competing interests

All authors declare that they have no competing interests.

## Authors’ contributions

ARNS conceived the study and conducted this work for her Master’s degree. LMKD conducted this work as part of her Master’s degree. MS and ECG made substantial contributions to the study design, analysis and interpretation of data. QALN, ECG and DDL carried out the molecular genetic studies, participated in the sequence alignment and drafted the manuscript. SMA and MJOT were involved in revising the manuscript for important intellectual content. MLG coordinated the work, supervised the drafting of the manuscript and contributed to the study design, analysis and interpretation of data. All authors read and approved the manuscript.
